# HPV E6/E7 mRNA test for the detection of high grade cervical intraepithelial neoplasia (CIN2+): a systematic review

**DOI:** 10.1186/s13027-020-0278-x

**Published:** 2020-02-07

**Authors:** Awoke Derbie, Daniel Mekonnen, Yimtubezinash Woldeamanuel, Xaveer Van Ostade, Tamrat Abebe

**Affiliations:** 1 Department of Medical Microbiology, College of Medicine and Health Sciences, Bahir Dar University, Bahir Dar, Ethiopia; 2 Centre for Innovative Drug Development and Therapeutic Trials for Africa (CDT-Africa), Addis Ababa University, Addis Ababa, Ethiopia; 3 Department of Health Biotechnology, Biotechnology Research Institute, Bahir Dar University, Bahir Dar, Ethiopia; 4 Department of Medical Microbiology, Immunology and Parasitology, School of Medicine, College of Health Sciences, Addis Ababa University, Addis Ababa, Ethiopia; 5 Laboratory of Protein Science, Proteomics and Epigenetic Signaling (PPES), Department of Biomedical Sciences, University of Antwerp, Antwerp, Belgium

**Keywords:** HPV E6/E7 mRNA test, Diagnostic performance, CIN2+

## Abstract

**Background:**

Genital infection with certain types of Human papillomavirus (HPV) is a major cause of cervical cancer globally. For early detection of premalignant dysplasia, evidences are coming out on the usefulness of HPV E6/E7 mRNA test as a potential tool compared with cytology and HPV DNA testing. Taking into account shortage of compiled data on this field, the aim of this systematic review was to describe the latest diagnostic performance of HPV E6/E7 mRNA testing to detect high grade cervical lesions (CIN2+) where by histology was taken as a gold standard.

**Methods:**

Articles published in English were systematically searched using key words from PubMed/Medline and SCOPUS. In addition, Google Scholar and the Google database were searched manually for grey literature. Two reviewers independently assessed study eligibility, risk of bias and extracted the data. We performed a descriptive presentation of the performance of E6/E7 mRNA test (in terms of sensitivity, specificity, negative and positive predictive values) for the detection of CIN2 + .

**Results:**

Out of 231 applicable citations, we have included 29 articles that included a total of 23,576 study participants (age range, 15–84 years) who had different cervical pathologies. Among the participants who had cervical histology, the proportion of CIN2+ was between 10.6 and 90.6%. Using histology as a gold standard, 11 studies evaluated the PreTect HPV Proofer, 7 studies evaluated the APTIMA HPV assay (Gen-Probe) and 6 studies evaluated the Quantivirus® HPV assay. The diagnostic performance of these three most common mRNA testing tools to detect CIN2+ was; 1) PreTect Proofer; median sensitivity 83%, specificity 73%, PPV 70 and NPV 88.9%. 2) APTIMA assay; median sensitivity 91.4%, specificity 46.2%, PPV 34.3% and NPV 96.3%. 3) Quantivirus®: median sensitivity 86.1%, specificity 54.6%, PPV 54.3% and NPV was at 89.3%. Further, the area under the receiver operating characteristics (AU-ROC) curve varied between 63.8 and 90.9%.

**Conclusions:**

The reported diagnostic accuracy implies that HPV mRNA based tests possess diagnostic relevance to detect CIN2+ and could potentially be considered in areas where there is no histology facility. Further studies including its cost should be considered.

## Background

According to the Global Cancer Statistics report of the year 2018, cervical cancer ranks 4th for both incidence and mortality with over 570,000 cases and 311,000 deaths worldwide [1]. The global burden of cervical cancer (close to 85%) occurs in developing countries, where it accounts for about 12% of all female cancers [1, 2]; and nine out of ten cervical cancer deaths disproportionately occur in developing countries [3–9].

About 95–99% of cervical cancer cases are associated with genital infection with High risk (HR)-HPV, which is the most common viral infection of the reproductive tract globally [10, 11] that most women are experiencing soon after they become sexually active [12]. Persistent infection with HR-HPV is the primary cause of cervical cancer and its precursor lesion, called the cervical intraepithelial neoplasia (CIN) [13, 14]. Next to cervical cancer, the HR-HPV has also been linked to a large proportion of other kinds of cancers (anus, vulva, vagina and penis) and a growing number of head and neck tumors [15–19]. So far, > 150 different HPVs have been characterized and completely sequenced. Of all types, about 40 are sexually transmitted and infect the genitalia [17, 20–23]. HPV types 16 and 18 are notably responsible for > 70% of all cervical cancer cases worldwide [15, 24, 25].

Cervical cancer is curable if detected at its early stage. Premature detection cervical lesion is valuable as it develops slowly preceding cervical cancer which typically takes over a period 10 years [26]. These precursor lesions called the cervical intraepithelial neoplasia (CINs) could be detected by a variety of methods [27]; the most frequently used one is cytology, but there are other alternative methods such as HPV DNA tests and visual inspection with acetic acid (VIA) [15], the latter being practiced primarily in resource limited settings [15]. However, cytology and HPV-DNA based tests are widely available in most developed nations [28, 29]. HPV DNA testing is being introduced in some countries as an adjunct to cytology (‘co-testing’) or as the primary screening test to be followed by a secondary test such as cytology or measurement of HR-HPV E6/E7 gene products [15, 30, 31].

Getting tools with reasonable diagnostic performance remains a challenge in the fight against cervical cancer globally. Given the inadequacy of existent methods, plus limitations in the use of both cytology and HPV DNA test (including but not limited to the sensitivity/specificity issues and the inability to indicate the risk of progression to cancer), there has long been interest in the development of new screening tools in cervical cancer [28, 29]. Many discovery approaches to find HPV associated cervical cancer screening biomarkers are currently underway, of which the HR-HPV *E6/E7 mRNA* test is a promising non-invasive biomarker for the detection of high grade cervical lesion (CIN2+) enabling detection of the HPV infection and simultaneously predicting the change of cervical lesions [32–40]. This is because continuous expression of E6/E7 oncogenes of HR-HPVs is necessary for the development and maintenance of the dysplastic phenotype [41].

It is known that the synergistic effect of the E6 and E7 proteins results in a disturbance of cell cycle regulation, apoptosis prevention, and the transformation and maintenance of neoplastic and dysplastic cells [42]. The overexpression of the E6 and E7 proteins, which inactivate the p53 and pRB respectively, can be detected by testing for the E6/E7 mRNA transcripts which is a potential biomarker (proxy indicator) for an increased risk of disease progression to the level of cancer [43–46]. The E6/E7 mRNA test can therefore help in avoiding aggressive procedures (biopsies and over-referral of transient HPV infections) as well as lowering patient’s anxiety and the follow-up period as well [34].

The detection of HR-HPV E6/E7 mRNA is being tested as the potential biomarker to elucidate the oncogenic role of HPV in cancer of the cervix and other kind of tumors in general [30, 35, 36, 39, 40, 42]. However, there is quite limited systematically reviewed data on the diagnostic role of different HPV E6/E7 mRNA tests for detection of CIN2+, using histology as a gold standard test. Specifically, the test performance of Aptima, Quantivirus and PreTect Proofer was assessed. The two tests (Aptima, Quantivirus) are able to detect the E6/7 mRNA from the same 14 HPV types, however, one of these tests (Aptima) is FDA approved and has a number of publications in the literature, whereas the other one (Quantivirus) has no clinical population based study published so far and only few entries in the web. The third test (PreTect Proofer) is only able to detect the E6/7 mRNA of five HPV types.

## Main text

### Methods

#### Protocol registration

In accordance with the PRISMA guidelines, our systematic review protocol was submitted to the International Prospective Register of Systematic Reviews (PROSPERO) and registered with a registration number ‘CRD42019123382’.

#### Eligibility criteria

Studies were selected based on the following criteria; *Study design*: we considered observational quantitative studies, like cross-sectional and cohort studies that reported the diagnostic performance of HPV E6/E7 mRNA assays for the detection of CIN2+. (CIN2+ refers to: histologically confirmed high grade lesions (CIN2, CIN3 and cancer)). *Participants*: We included studies that included women (cervical sample) having different kinds of cervical pathology. *Language and publication*: we included peer-reviewed journal articles published in English in the period of 2011 to 14 Jan 2019 with the outcome of interest reported in different countries.

#### Information sources and search strategy

This review was done following the Preferred Reporting Items for Systematic Reviews and Meta-Analysis Protocol (PRISMA) guideline [47]. Research papers were systematically searched in PubMed/Medline and SCOPUS using key words by combing using Boolean operator. Manual search from Google scholar and Google databases was also performed for grey literature; the last search was done on 14th of Jan, 2019. The reference lists of retrieved articles were probed (forward and back ward searching) to identify articles that were not retrieved from databases and our manual search. The first two authors; AD and DM searched the articles independently.

The domains of the search terms were Human Papillomaviruses, HPV, E6/E7 mRNA, and Cervical Intraepithelial Neoplasia. We combined Human Papillomaviruses and HPV with the Boolean operator “OR”, and the result was combined with the other terms with “AND”. Full search strategy for the two databases is annexed in Additional file 1.

#### Study selection

Research papers that reported the type of HPV E6/E7 mRNA diagnostic performance for the detection of CIN2+ were included. Searched articles were directly imported and handled using EndNote X5 citation manager (Thomson Reuters, New York, USA). Based on the PRISMA protocol, duplicated articles were excluded and the titles and abstracts of the remaining papers were screened independently for inclusion in full text evaluation by the first two authors. Differences between the reviewers were resolved through discussion.

#### Data collection process and data items

Data such as the name of the first author, year of publication, country where the study was conducted, age group of the study participants, type of women included in the study, CIN profile of the study participants, the proportion of CIN2+, type of HPV E6/E7 mRNA test used, the positivity rate of the mRNA test and its diagnostic performance (in terms of sensitivity, specificity, Positive Predictive value (PPV) and Negative Predictive Value (NPV)) were extracted from the included articles.

#### Quality appraisal

To assess the risk of bias, the Critical Appraisal Skills Programme (CASP) tool [48] that developed to evaluate studies of diagnostic test accuracy was independently used by the first two authors. Of the twelve criterion of the tool, we eliminated three items because we felt that their scoring is difficult and rating the items would be more of subjective. Assessment of quality results was categorized but not summarized into a score as the method has little validity [48].

#### Data synthesis

The extracted data were fed into a Microsoft Excel and presented in terms of 1) CIN profile of the study subjects, 2) the proportion of CIN2+, 3) the proportion of mRNA test result 4) diagnostic performance of mRNA test to detect CIN2+ (sensitivity, specificity, PPV and NPV). Owing to the presence of a large amount of clinical heterogeneity of the included articles and as most of the papers didn’t present the 2 × 2 table, pooling of the diagnostic performance of mRNA tests was not possible. Instead, we performed a descriptive presentation of these elements (using range) to compile a best evidence synthesis for E6/E7 mRNA HPV testing in the detection of CIN2+. A systematic narrative synthesis was provided in which summary results were presented using text, table and figures. Descriptive statistics, such as: simple counts, ranges and percentages were used to describe the findings.

## Results

### Search results

**Fig. 1:**
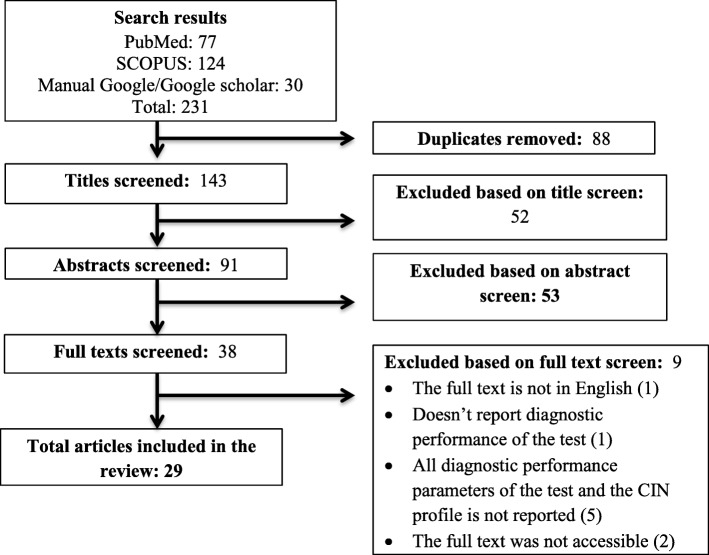
The PRISMA flow diagram of literature selection

From the systematically searched databases and other sources, a total of 231 articles were retrieved and sequentially screened. After removing 88 duplicates, the 143 were screened by title then 52 were removed. Consequently, 53 were removed by abstract and 9 by full text with justifiable reasons. Finally, a total of 29 studies met our inclusion criteria. Screening was based on the PRISMA flow chart which was adapted from the PRISMA guidelines [49] (Fig. 1).

### Characteristics of the included studies

The characteristic of the included articles is summarized in (Table 1 and Table 2). The studies were reported from 13 different countries in Europe, Asia and the United States of America. We didn’t find articles reported in Latin America and Africa, in particular. The number of participants in each included study varied from 60 to 9451. Seven studies did not report the age of the study participants. The included studies employed a total of 23,576 study participants (age range, 15–84 years) who had different cervical pathologies. The studies were of varying methodological quality, and were predominately performed in a secondary screening setting (i.e women or cervical samples were subjected to HPV E6/E7 mRNA testing secondary to having positive cervical cytology and/or positive HPV DNA test). Similarly, diverse histological findings were reported ranging from normal histology to cervical cancer (Table 1).

**Table 1:** Characteristics of the included studies, 2011–18

Author (s), Year	Country	#Partici-pants	Age group, median	Study population characteristics	Histology profiles
Ratnam, 2011	Canada	1418	15–80, 30.6	Women referred to colposcopy and those routinely screened	Normal = 651, CIN1 = 366, CIN2 = 120, CIN2 + =401, CIN3 = 281
Waldstrom, 2011	Denmark	405	16–65, 32	Three years archived liquid-based samples, all with a diagnosis of LSIL	CIN2 + =67, CIN3 + =33
Fan, 2018	China	192	No data	Patients with abnormal cytology results and/or high-risk HPV infection	CIN1- = 41, CIN1 = 54, CIN2 = 28, CIn3 = 53, cancer = 16
Han,2018	China	197	No data	Women with abnormal cytological or HPV test results	CIN1- = 80, CIN1 = 16, CIN2/3 = 50, Caner = 51
Binnicker,2014	USA	350	No data	Residual specimens with a cytologic result of ≥ atypical squamous cells of undetermined significance (ASC-US)	CIN2 + =81, CIN3 + =41
Broccolo,2013	Italy	308	20–65	A retrospective study on cervical cytology specimens	Normal = 70, CIN1 = 159, CIN2 + =79
Waldstrom, 2012	Denmark	235	30–69, 42.2	Women with ASC-US cytology	Normal = 86, CIN1 = 35, CIN2 = 21, CIN3 = 26, Cancer = 1
Li,2017	China	318	No data	Women with ASCUS cytology attending routine outpatient primary cervical screening	Normal = 169, CIN1 = 74, CIn2 = 40, CIN3 = 16, cancer = 19
Benevolo,2011	Italy	464	17–78, 32	Hybrid Capture 2 (HC2)-positive patients, who underwent colposcopy	<CIN2 = 49, CIN2 + =49, SCC = 9
Wang, 2019	Korea	563	20–84, no	Liquid-based cytology samples	Normal = 51, CIN1 = 75, CIN2 = 16, CIN3 = 40, cancer = 38
Liu, 2014	China	335	No data	Women who underwent outpatient hospital-based gynecological screening	Normal = 30, CIN1 = 5, CIN2 = 1,CIn3 = 15, Cancer 41
Iftner, 2015	Germany	9451	30–60	Women undergoing routine cervical screening	<CIN2 = 513, CIN2 = 47, CIN3 + =43
Sorbye, 2011	Norway	520	25–69	Post-colposcopy follow-up of women with negative or low-grade biopsy	CIN2- = 396, CIN2 + = 124
Duvlis, 2015	Macedonian	413	19–78	Women that come for cervical cancer screening	Normal = 10, CIN1 = 22, CIN2 = 20, CIN3 = 9
Castro, 2013	Spain	165	18–75, 34.8	Women with endocervical samples harboring HPV 16 and/or 18 DNA	CIN2- = 93, CIN2 + = 72
Pierry, 2012	USA	246	19–75	Women in a higher risk urban screening population	Normal = 137, CIN1 = 64, CIN2 = 25, CIN3 = 20
Alaghehbandan, 2013	Canada	1360	15–80, 30.7	women with a history of abnormal cytology referred to colposcopy	Normal = 635, CIN1 = 345, CIN2 + =380, Cancer = 10
Clad, 2011	Germany	424	No data	Women referred to colposcopy due to an abnormal Pap smear	Normal = 108, CIN1 = 64, CIN2 = 89, CIN3 = 150, Cancer = 13
Varnai, 2018	Germany	66	21–66, 34.6	Office based screening population who were HPV-DNA positive for at least one of the following HR- HPV types: HPV 16, 18, 31, 33 and 45	Normal = 6, CIN2 = 5, CIN3 = 22
Coquillard, 2011	USA	2049	No data	Women both low and high risk	Normal = 1694, CIN1 = 78 CIN2 + =73
Shen, 2013	China	272	16–77, 37	Women with abnormal colposcopy	CIN1- = 80, CIN2 = 6, CIN3 + =25
Li, 2016	China	186	22–68	Patients underwent colposcopy	Normal = 32, CIN1 = 51, CIN2/3 = 71, cancer = 32
Liu, 2017	China	380	> 30	Women who were associated with high risk of cervical virus infection	Normal = 159, CIN1 = 68, CIN2 = 74, CIN3 = 68, cancer = 11
Benevolo, 2011	Italy	1610	18–83, 39.5	Retrospective analysis of women underwent an E6/E7 mRNA test on cervical samples	Normal = 74, CIN1 = 282, CIN2 = 120, CIN3 = 86, cancer = 24
Liu, 2018	Japan	171	22–76, 33	Women with pathologically-diagnosed CIN or cervical carcinoma	CIN1 = 16, CIN2 = 33, CIN3 = 83, SCC = 39
Persson, 2014	Sweden	219	23–60, 32	HR-HPV-positive women diagnosed with atypical squamous cells of undetermined significance (ASCUS) and low-grade squamous intraepithelial lesions (LSIL)	Normal = 56, CIN1 = 69, CIn2 = 37, CIN3+ 36
Sorbye, 2011	Norway	297	25–69	Women with ASC-US and LS	CIN1- = 53, CIN2 + =69
Andersson, 2011	Sweden	204	21–79, 32	Women who were undergoing gynecological screening or had been admitted to a referral center	Normal = 45, CIN1 = 33, CIn2 = 28, CIN3 = 31, Cancer 28
Oliveira, 2013	Portugal	554	18–73, 33	Women were referred for opportunistic screening and for evaluation of HPV associated lesions	Normal = 131, CIN1 = 128, CIn2 = 129, CIN3 = 146, Cancer = 14

**Table 2:** The proportion of HPV E6/E7 mRNA test result and its diagnostic performance of against histology, 2011–18

Author (S)	Prevalence of CIN2+, n (%)	HPV DNA test	HPV E6/E7 mRNA test	mRNA test positivity rate, n(%)	Performance of E6/E7 mRNA test				
					Sen%	Spe%	PPV%	NPV%	AU-ROC%
Ratnam, 2011	28.30%	Hybrid Capture 2 (HC2)	Aptima	964 (68%)	96.3	46.2	40.0	96.7	
Waldstrom, 2011	67(16.5)	none	Aptima	271 (67%)	92.5	38.2	22.9	96.3	
Fan, 2018	97 (50.5)	HC2	Fluorescence in situ hybridization (FISH)	no data	91.5	81.6	82.7	90.9	90.9
Han,2018	101 (51.3)	No data	No data	No data	85.2	66.7	72.9	81	74.95
Binnicker,2014	81(23.1)	HC2	APTIMA	230 (65.7%)	91.4	42	32.1	94.2	
Broccolo,2013	79 (25.6)	Quantitative real-time PCR	PreTect HPV-Proofer	115 (37.3)	77	81.7	66.9	88	
Waldstrom, 2012	48 (55.8)	Linear Array	Aptima	103 (31.7%)	87.5	78	40.8	97.3	
Li,2017	85 (26.7)	HC2	QuantiVirus HPV E6/E7 RNA 3.0;	177 (70.2)	88.2	36.4	33.9	89.3	68.5
Benevolo,2011	49 (10.6)	HC2	Pretect HPV-Proofer	165 (36%)	72	73	39	92	
Wang, 2019	104 (47.3)	HPV DNA chip test	Optimygene HR-HPV RT-qDx assay,	219 (38.9)	85.9	82.5	78.2	87.4	
Liu, 2014	57 (61.9)	No	Quantivirus®	135 (40.3)	71.9	74.3	82	61.9	78
Iftner, 2015	90 (14.9)	HC2	Aptima	464 (4.9%)	87.8	96.1	21.1	99.8	
Sorbye, 2011	124 (23.8)	No	PreTect HPV-Proofer	52 (27.1)	89.1	92.5	77.3	96.4	
Duvlis, 2015	29 (47.5)	No	NucliSENS-EasyQ® (PreTect Proofer)	74 (17.9)	93.1	50	62	88.9	
Castro, 2013	72 (43.6)	Nested PCR	NucliSENS-EasyQ® (PreTect Proofer)	96 (58.2)	84.1	80	90.9	88.9	
Pierry, 2012	45 (18.3)	none	(HPV OncoTect		89	92	71		
Alaghehbandan, 2013	380 (27.9)	HC2	PreTect HPV-Proofer	525 (38.6)	76.1	68.7	55	89.1	
Clad, 2011	252 (59.4)	HC2	Aptima	274 (65)	91.7	75			
Varnai, 2018	27 (81.8)	PCR-based typing	PreTect HPV-Proofer	38 (58)	95	55	81	86	
Coquillard, 2011	73 (28.1)	HC2	Quantivirus®	No data	84	85	78	–	
Shen, 2013	31 (27.9)	HC2	Quantivirus®	200 (73.5)	82.4	15.5	22.2	75	63.8
Li, 2016	103 (55.4)	HC2	Quantivirus®	138 (74.2)	91.3	47	68.1	81.3	75.9
Liu, 2017	153 (40.3)	none	Quantivirus®	275 (72.4)	93.4	62.1	40.5	97.3	
Benevolo, 2011	230 (39.2)	HC2	PreTect HPV-Proofer	166 (10.3)	83	45	80	91	
Liu, 2018	155 (90.6)	SepaGene kit	RT-PCR assay	95 (55.6)	55	50	91	77	
Persson, 2014	73 (36.9)	Linear Array Genotyping	APTIMA	162 (74)	78.1	25	36.5	67.4	
Sorbye, 2011	69 (23.2)	None	PreTect HPV-Proofer	97 (32.7)	94.2	86	67	98	
Andersson, 2011	87 (56.1)	None	Real-time PCR	No data	91	68	67	91	
Andersson, 2011	87 (56.1)	None	PreTect HPV-Proofer	No data	75	77	70	81	
Oliveira, 2013	295 (53.8)	HC2	NucliSENS EasyQ® (PreTect Proofer)	305 (55.1)	79.3	72.6	76.7	75.5	

Among those participants who had cervical histological examination, the proportion of CIN2+ was between 10.6 and 90.6%. The reported proportion of positive mRNA test varied from 10.3 to 74.2%. With regard to the type of HPV E6/E7 test methods, 11 studies evaluated the PreTect Proofer, 7 studies evaluated the APTIMA HPV assay, 6 studies evaluated the Quantivirus® HPV assay and the remaining studies each evaluated Fluorescence in situ hybridization (FISH), Optimygene HR-HPV RT-qDx assay, HPV OncoTect, RT-PCR assay based on consensus primers and real-time PCR assay. A study [50] evaluated both real-time PCR assay and PreTect Proofer. On top of this, about 13 studies compared HPV E6/E7 mRNA testing to the Hybrid Capture 2 (HC2) DNA test (Digene/Qiagen), two studies compared the mRNA test to Linear Array (Roche Diagnostics), two studies compared the mRNA test to DNA PCR testing, one study used SepaGene kit, one study used HPV DNA chip test and one study used QuantiVirus®HPV DNA Diagnostic Kit. The rest didn’t compare the mRNA test with HPV DNA test. The agreement between HPV-DNA and HPV-E6/E7 mRNA test in the detection of cervical lesions (which varied from 77.6 to 92.5%) was reported by only 8 studies [39, 51–57] (Table 2).

### Risk of bias

Ratings of the study quality for each of the nine domain-based critical appraisal skills program (CASP) [48] criteria to make sense of a diagnostic test study are presented in Additional file 2. The risk of bias for each individual domain was rated as ‘Yes’, ‘No’ or ‘Can’t tell’. The assessment of quality results was categorized not scored otherwise. The mRNA test predictive values were not reported by Clad et al. [56]. Otherwise we felt that all the included studies had no major methodological uncertainties.

### Diagnostic performance of HPV E6/E7 mRNA testing to detect CIN2+

Due to the difference in clinical presentation of subjects (considerable clinical heterogeneity of the study participants by the included studies), pooling the diagnostic performance data was not possible. In its place, we compiled a best evidence synthesis for HPV E6/E7 mRNA HPV testing to detect high grade cervical lesions (CIN2+) using descriptive statistics.

The diagnostic performance of the three most common mRNA testing tools as compared with histologically confirmed high-grade cervical intraepithelial neoplasia (CIN2+) as an endpoint was as follows; 1) PreTect Proofer; median sensitivity 83%; ranged 72–95%, median specificity 73%; range 45–92.5%, median PPV 70%; range 39–90.9%, median NPV 88.9%; range 81–98%, 2) APTIMA assay; median sensitivity 91.4%; range 78.1–96.3%, median specificity 46.2%; range 25–96.1%, median PPV 34.3%; range 21–40.8% and median NPV 96.3%; range 67.4–99.8% and 3) Quantivirus®: median sensitivity 86.1%; range 71.9–93.4%, median specificity 54.6%; range 36.4–85%, median PPV 54.3%; range 22.2–82%, median NPV 89.3%; range 61.9–97.3%.

Only six studies [51, 58–62] reported the area under the receiver operating characteristic (ROC) curve for diagnosing high-grade cervical lesions (CIN2+) by HPV E6/E7 mRNA tests considering histology as a gold standard. The reported area under the curve varied from 63.8 to 90.9% (the curve was to the left of the diagonal) with overall mRNA test median sensitivity, 87.7%, range 55–96.3%; median specificity 70.7%, range 25.5–96.1%; median PPV 67%, range 21.1–91%; and median NPV 83.7%, range 77–99.8% (Table 2).

## Discussion

There is a previous review on the performance of HPV mRNA test by Burger et al. [63] which was published in 2010. Our review is an update of the latest knowledge on the test performance of HPV E6/E7 mRNA test, compiled from articles published since 2011. We included double number of studies with varying methodological quality but our finding is in line with this review. Hence, together with the previous review [63], our finding would be considered for further large scale studies to generate bold data on the clinical applicability of the test.

In the present review, women were tested for the HPV E6/E7 mRNA predominately secondary to having positive cervical cytology and/or positive HPV DNA test. Similarly, diverse histological findings were reported ranged from normal histology to cervical cancer. The HPV E6/E7 mRNA tests detected 10.3 to 74.2% proportions of cervical lesions from participants who had different level of cervical pathology. A study by Liu et al. showed that the positivity rate of HPV E6/E7 mRNA test increased with the severity of cytological or histological findings [60] in which all samples with high-grade lesions were positive for the HPV mRNA test as described previously [40]. All grades of histological findings were reported in this review. Specifically, the proportion of CIN2+ was between 10.6 and 90.6%. This reflects the diverse spectrum of cervical pathologies of the participants employed by the included articles. Due to this heterogeneity of the employed participants by the included studies, the results of the HPV E6/E7 mRNA test performance have limited generalizability and conclusion should be considered with caution. Moreover, extreme diagnostic results were also reported, for example, the test specificity reported by Sorbye et al. [64] was at (92.5%) and Persson et al. [65] was at (25%). Similarly, extreme positive predicative values (PPVs) of the mRNA test were reported by Iftner et al. at 21.1% [55] and Liu et al. at 91% [66] in which the disparity might be resulted from a difference in the type of included study participants who had different cervical pathologies.

Our review report proved that the E6/E7 mRNA tests have diagnostic relevance to detect CIN2+, especially with good test specificity. This is consistent with a previous study [63]. Recent evidences also showed that detection of the HPV E6/E7 mRNA transcripts may provide a higher specificity for the detection of high grade cervical lesions, since the oncogenic potential of HPV infection depends on the over expression of these two transcripts [57] but the test methods lack either detection of all high-risk HPV genotypes (like, PreTect HPV-Proofer) or the capacity to specify the detected genotypes (like, APTIMA) [50]. In general a high specificity and NPV of the E6/E7 HPV mRNA test [35] can be translated into a low referral rate for colposcopy [61], which is not commonly available, particularly in resource limited settings.

There are several HPV testing kits/products available [67]. In our review, studies used the following kind of HPV E6/E7 mRNA testing products; PreTect Proofer (NorChip AS)/NucliSENS-EasyQ® (Biomerieux), APTIMA HPV assay, Quantivirus® HPV assay, Fluorescence in situ hybridization (FISH), Optimygene HR-HPV RT-qDx assay, HPV OncoTect, RT-PCR assay based on consensus primers and real-time PCR assay. The first two methods are commonly utilized tests in the field of HPV mRNA test; hence in this review out of 29 included studies 18 used these two methods.

Taking histology confirmed CIN 2+ as the disease endpoint to assess the clinical performance of the test, the sensitivity of APTIMA at (78.1–96.3%) was better than the PreTect HPV-Proofer (72 to 95%) and Quantivirus® (71.9–93.4%). However, in terms of specificity (45–92.5%) the PreTect HPV-Proofer was much better than these two tests. This might partly because, the PreTect HPV-Proofer detects only the mRNA of the five most common HPV types (HPV16, 18, 31, 33, and 45) hence is more specific than the APTIMA and Quantivirus® assays which detect mRNA of more genotypes. Targeting more genotypes makes the latter two methods rather more sensitive than the PreTect Proofer [50]. Our review result is in line with this argument. On top of this, the reported AU-ROC curve by six of the articles [51, 58–62] varied from 63.8 to 90.9% which reflects the usefulness of the test in discriminating women having CIN2 + .

In our review 8 studies [39, 51–57] reported the agreement between HPV-E6/E7 mRNA and HPV-DNA testing for the detection of cervical lesions at 77.6–92.5%. Compared to DNA-based tests (the most common one was Digene Hybrid Capture 2 HPV DNA test) which indicate only the presence or absence of the virus, detecting HPV E6/E7 mRNAs gives more insight into viral activity and by implication, clinical relevance (correlate better with the severity of the lesion). Hence, the latter is a potential marker for the identification of women at risk of developing cervical carcinoma. Our review support the argument that the HPV E6/E7 mRNA assay could overcome the shortcoming of low specificity of DNA assays for clinical detection of high-grade cervical lesions [35, 68–75].

The Burger et al. [63] review on HPV mRNA test for the detection of cervical intraepithelial neoplasia, the reported sensitivities varied between 41 and 86% and 90–95% for the PreTect Proofer/Easy Q and APTIMA assay, respectively. Similarly, the reported specificities vary from 63 to 97% and 42–61% for the PreTect Proofer/Easy Q and APTIMA assay, respectively. These figures are almost in line with our reports. It is good to note that a higher specificity, especially in a triage setting may reduce the number of women that would be subjected to unnecessary conizations and expensive follow-up [63, 71] and patients psychologic burden associated with repeated HPV-DNA testing [72]. However, an individual study by Shen et al. does not support the use of this assay in screening for cervical cancer prevention alone [61].

A study by Yang et al. also showed that a woman tested positive for HPV E6/E7 mRNA had a higher risk of progressing to high grade cervical lesions. Whereas, women with a negative HPV E6/E7 mRNA can increase follow-up interval, by comprehensively considering their situation, thus, avoiding unnecessary colposcopy and reducing the rate of colposcopy and biopsy [76]. In our review the overall mRNA test performance in terms of negative predicative value (NPV) was at (77–99.8%) which is in line with the above statement in which mRNA test had a very good negative prediction of women for high grade cervical lesions if they get tested negative. These help patients from unnecessary follow-up including the expensive colposcopy, diagnostic and therapeutic procedures [64]. In contrary, women tested positive in the HPV E6/E7 mRNA test have a greater risk of malignant progression of cervical lesions and therefore needs more care and earlier check-ups [77].

### Limitations

This systematic review presents the latest developments in the field of HPV E6/E7 mRNA test accuracy. We have included relatively adequate number of articles published in different countries employing a large number of study participants. However, our review result should be interpreted in light of a couple of shortcomings. The main drawback of our review is the lack of studies that employed similar and well-defined population with same cervical pathology characteristics. Hence, the review was suffered from heterogeneity of the studies and was not possible to pool the performance of the mRNA tests. Moreover, the decisive aim of cervical cancer screening is to detect cervical lesions that will develop into cancer. However, the use of histologically confirmed CIN2+ endpoint when evaluating mRNA accuracy represents a challenge because of the regression (false positive) or progression (false negative) of many confirmed lesions [63]. Confining our inclusion criteria to include only articles published in English languages may introduce missing of relevant studies and reduced the precision of our results.

## Conclusions

The reported test performance and the receiving operating characteristics curves implies that HPV E6/E7 mRNA testing has a diagnostic relevance to detect CIN2+ and could be considered in areas where there is no histological test facility. Further studies including its cost should be considered. Moreover, future research in the field should emphasis on the clinical translation (utility) of HPV E6/E7 mRNA tests using large consecutive cohorts of women, including participants from developing nations, representing a well-defined population for a specific type of cervical pathology.

## Supplementary information

**Additional file 1.** Search strategy

**Additional file 2.** Risk of bias summary result: review authors’ judgements about each risk of bias item for included studies, 2011–18

## Data Availability

All the generated data in this review are included in the manuscript.

## Abbreviations

ACS: American Cancer Society
ASCUS: Atypical squamous cells of undetermined significance
CI: Confidence Interval
CIN: Cervical Intra Neoplasia
DNA: Deoxyribonucleic Acid
E6/7: Early protein 6/7
HIV: Human Immunodeficiency virus
HPV: Humanpapillom Virus
HR-HPV: High-risk Humanpapilloam virus
Kb: Kilo base pair
L1/2: Late Protein ½
LCR: Long Control Region
mRNA: messenger ribonucleic acid
NA: Nucleic Acid
NPV: Negative Predictive Value
ORF: Open Reading Frame
Pap: Papaniculo test
PCR: Polymerase Chain Reaction
pHR: Probable High Risk
PPV: Positive Predictive Value
pRb: Retinoblastoma Protein
qPCR: quantitative Polymerase Chain Reaction
ROC: *Receiver Operating Characteristic curve*
SCC: Squamous Cell Carcinoma
VIA: Visual Inspection with Acetic acid
WHO: World Health Organization

## References

[1] Bray, F., et al., Global cancer statistics 2018: GLOBOCAN estimates of incidence and mortality worldwide for 36 cancers in 185 countries. CA Cancer J Clin, 2018. 0(0). 10.3322/caac.21492 30207593

[2] WHO. Integrated Africa Cancer Factsheet Focusing on Cervical Cancer. 2014 [cited 2018 3Oct]; Available from: http://www.who.int/pmnch/media/events/2014/africa_cancer_factsheet.pdf.

[3] WHO, Human Papil-lomavirus and Related Cancers in World (2010). Summary Report 2010.

[4] Melek A, Bayik A (2011). Visual inspection with acetic acid in cervical Cancer screening. Cancer Nurs.

[5] Sharma Garima, Dua Pradeep, Agarwal Subhash (2014). A Comprehensive Review of Dysregulated miRNAs Involved in Cervical Cancer. Current Genomics.

[6] WHO. Cervical Cancer: Estimated Incidence, Mortality and Prevalence Worldwide in 2012. 2012 [cited 2018 30 Jan]; Available from: http://globocan.iarc.fr/old/FactSheets/cancers/cervix-new.asp.

[7] Agency, C.S (2011). Ethiopian demographic health survey.

[8] WHO. Human papillomavirus (HPV) and cervical cancer. WHO Media center June 2016 [cited 2018 1 Feb]; Available from: http://www.who.int/mediacentre/factsheets/fs380/en/.

[9] WHO. Latest global cancer data. 2018 12 Sep 2018 [cited 2018 3 Oct]; Available from: http://www.who.int/cancer/PRGlobocanFinal.pdf.

[10] WHO, Guidelines for screening and treatment of precancerous lesions for cervical cancer prevention, 2013: World Health Organization, 20 avenue Appia, 1211 Geneva 27, Switzerland. 24716265

[11] Kambouris, M., V. Chini, and A. Daskalaki. HPV Detection and Genotyping Using the Luminex xMAP Technology. 2010 [cited 2018 15 Nov]; Available from: http://www.irma-international.org/viewtitle/40440/.

[12] Maine D, Hurlburt S, Greeson D (2011). Cervical Cancer prevention in the 21st century: cost is not the only issue. Am J Public Health.

[13] Santos-Lopez G (2015). General aspects of structure, classification and replication of human papillomavirus. Rev Med Inst Mex Seguro Soc.

[14] WHO. Human papillomavirus (HPV) and cervical cancer. 2016 [cited 2018 13 April]; Available from: http://www.who.int/mediacentre/factsheets/fs380/en/.

[15] Bruni, L., et al. ICO/IARC Information Centre on HPV and Cancer (HPV Information Centre). Human Papillomavirus and Related Diseases in the World. Summary Report 2017. 2017 [cited 2018 13 April]; Available from: http://www.hpvcentre.net/statistics/reports/XWX.pdf.

[16] Chow LT, Broker TR, Steinberg BM (2010). The natural history of human papillomavirus infections of the mucosal epithelia. Apmis.

[17] Doorbar J (2012). The biology and life-cycle of human papillomaviruses. Vaccine.

[18] Wallace NA, Galloway DA (2015). Novel functions of the human papillomavirus E6 Oncoproteins. Annu Rev Virol.

[19] Centre, I.I.I.C.o.H.a.C.H.I. Human Papillomavirus and Related Diseases Report. 2017 [cited 2018 28 Sep ]; Available from: http://www.hpvcentre.net/statistics/reports/XWX.pdf.

[20] Münger K (2004). Mechanisms of human papillomavirus-induced Oncogenesis. J Virol.

[21] Williams VM (2011). HPV-DNA integration and carcinogenesis: putative roles for inflammation and oxidative stress. Futur Virol.

[22] Kajitani N (2012). Productive lifecycle of human papillomaviruses that depends upon squamous epithelial differentiation. Front Microbiol.

[23] Narisawa-Saito M, Kiyono T (2007). Basic mechanisms of high-risk human papillomavirus-induced carcinogenesis: roles of E6 and E7 proteins. Cancer Sci.

[24] Kim, G. Harald zur Hausen's Experiments on Human Papillomavirus Causing Cervical Cancer (1976–1987): Embryo Project Encyclopedia (2017-03-09). 2017 [cited 2018 23 Oct]; Available from: https://embryo.asu.edu/pages/harald-zur-hausens-experiments-human-papillomavirus-causing-cervical-cancer-1976-1987.

[25] Bouvard V (2009). A review of human carcinogens--Part B: biological agents 2009. Lancet Oncol.

[26] Leto M (2011). Human papillomavirus infection: etiopathogenesis, molecular biology and clinical manifestations. An Bras Dermatol.

[27] García DA (2011). Highly sensitive Detection and genotyping of HPV by PCR multiplex and Luminex Technology in a Cohort of Colombian women with abnormal cytology. The open virology journal.

[28] Sahasrabuddhe VV, Luhn P, Wentzensen N (2011). Human papillomavirus and cervical cancer: biomarkers for improved prevention efforts. Future Microbiol.

[29] Tian, Q., et al., MicroRNA Detection in Cervical Exfoliated Cells as a Triage for Human Papillomavirus–Positive Women: J Natl Cancer Inst. 2014 Sep;106(9):dju241. doi:10.1093/jnci/dju241. 10.1093/jnci/dju241 PMC4188123 25190727

[30] Wang HY (2015). Diagnostic performance of HPV E6/E7 mRNA and HPV DNA assays for the Detection and screening of oncogenic human papillomavirus infection among woman with cervical lesions in China. Asian Pac J Cancer Prev.

[31] Cuschieri K, Wentzensen N (2008). Human papillomavirus mRNA and p16 detection as biomarkers for the improved diagnosis of cervical neoplasia. Cancer Epidemiol Biomark Prev.

[32] Fontecha N (2017). RNA extraction method is crucial for human papillomavirus E6/E7 oncogenes detection. Virol J.

[33] Mariano VS (2016). A Low-Cost HPV Immunochromatographic Assay to Detect High-Grade Cervical Intraepithelial Neoplasia. PLoS One.

[34] Duvlis S (2015). HPV E6/E7 mRNA versus HPV DNA biomarker in cervical cancer screening of a group of Macedonian women. J Med Virol.

[35] Zhao X (2014). Comparative study of HR HPV E6/E7 mRNA and HR-HPV DNA in cervical cancer screening. Zhonghua Yi Xue Za Zhi.

[36] Perez Castro S (2013). Human papillomavirus (HPV) E6/E7 mRNA as a triage test after detection of HPV 16 and HPV 18 DNA. J Med Virol.

[37] Cattani P (2009). Clinical performance of human papillomavirus E6 and E7 mRNA testing for high-grade lesions of the cervix. J Clin Microbiol.

[38] Lie AK, Kristensen G (2008). Human papillomavirus E6/E7 mRNA testing as a predictive marker for cervical carcinoma. Expert Rev Mol Diagn.

[39] Varnai AD (2008). Predictive testing of early cervical pre-cancer by detecting human papillomavirus E6/E7 mRNA in cervical cytologies up to high-grade squamous intraepithelial lesions: diagnostic and prognostic implications. Oncol Rep.

[40] Fontecha N (2016). Assessment of human papillomavirus E6/E7 oncogene expression as cervical disease biomarker. BMC Cancer.

[41] Johansson H (2015). Presence of High-Risk HPV mRNA in Relation to Future High-Grade Lesions among High-Risk HPV DNA Positive Women with Minor Cytological Abnormalities. PLoS One.

[42] Ren C (2018). Diagnostic performance of HPV E6/E7 mRNA assay for detection of cervical high-grade intraepithelial neoplasia and cancer among women with ASCUS Papanicolaou smears. Arch Gynecol Obstet.

[43] Alaghehbandan R (2013). Performance of ProEx C and PreTect HPV-proofer E6/E7 mRNA tests in comparison with the hybrid capture 2 HPV DNA test for triaging ASCUS and LSIL cytology. Diagn Cytopathol.

[44] Ratnam S (2010). Clinical performance of the PreTect HPV-proofer E6/E7 mRNA assay in comparison with that of the hybrid capture 2 test for identification of women at risk of cervical Cancer. J Clin Microbiol.

[45] Ratnam S (2011). Aptima HPV E6/E7 mRNA test is as sensitive as hybrid capture 2 assay but more specific at detecting cervical Precancer and Cancer. J Clin Microbiol.

[46] Chambers G (2014). Assessing the detection of human papillomavirus late mRNA in liquid base cytology samples for risk stratification of cervical disease. J Med Virol.

[47] Shamseer, L., et al., Preferred reporting items for systematic review and meta-analysis protocols (PRISMA-P) 2015: elaboration and explanation. Bmj, 2015. 2(350). 10.1136/bmj.g7647 25555855

[48] CASP. Critical Appraisal Skills Programme. 2018 26 Jan 2019]; Available from: https://www.unisa.edu.au/contentassets/72bf75606a2b4abcaf7f17404af374ad/2a-casp_cohort_tool.pdf.

[49] Moher D (2009). Preferred reporting items for systematic reviews and meta-analyses: the PRISMA statement. PLoS Med.

[50] Andersson E (2011). Type-specific human papillomavirus E6/E7 mRNA Detection by real-time PCR improves identification of cervical Neoplasia. J Clin Microbiol.

[51] Fan Y, Shen Z (2018). The clinical value of HPV E6/E7 and STAT3 mRNA detection in cervical cancer screening. Pathol Res Pract.

[52] Broccolo F (2013). Comparison of oncogenic HPV type-specific viral DNA load and E6/E7 mRNA detection in cervical samples: results from a multicenter study. J Med Virol.

[53] Waldstrom M, Ornskov D (2012). Comparison of the clinical performance of an HPV mRNA test and an HPV DNA test in triage of atypical squamous cells of undetermined significance (ASC-US). Cytopathology.

[54] Wang H-Y, Kim H, Park KH (2019). Diagnostic performance of the E6/E7 mRNA-based Optimygene HR-HPV RT-qDx assay for cervical cancer screening. Int J Infect Dis.

[55] Iftner T (2015). Head-to-head comparison of the RNA-based aptima human papillomavirus (HPV) assay and the DNA-based hybrid capture 2 HPV test in a routine screening population of women aged 30 to 60 years in Germany. J Clin Microbiol.

[56] Clad A (2011). Performance of the Aptima high-risk human papillomavirus mRNA assay in a referral population in comparison with hybrid capture 2 and cytology. J Clin Microbiol.

[57] Oliveira A, Verdasca N, Pista A (2013). Use of the NucliSENS EasyQ HPV assay in the management of cervical intraepithelial neoplasia. J Med Virol.

[58] Han L (2018). Clinical value of human papillomavirus E6/E7 mRNA Detection in screening for cervical Cancer in women positive for human papillomavirus DNA or. Clin Lab.

[59] Li Y (2017). Detection of cervical intraepithelial neoplasia with HPVE6/E7 mRNA among women with atypical squamous cells of unknown significance. Int J Gynaecol Obstet.

[60] Liu TY (2014). Diagnostic validity of human papillomavirus E6/E7 mRNA test in cervical cytological samples. J Virol Methods.

[61] Shen Y (2013). Quantivirus(R) HPV E6/E7 RNA 3.0 assay (bDNA) is as sensitive, but less specific than hybrid capture 2 test. J Virol Methods.

[62] Li J (2016). Risk evaluation of cervical cancer progress by screening human papillomavirus DNA, E6/E7 mRNA and protein, and cell free ferrous protoporphyrin. Int J Clin Exp Med.

[63] Burger EA (2011). HPV mRNA tests for the detection of cervical intraepithelial neoplasia: a systematic review. Gynecol Oncol.

[64] Sorbye SW (2011). Triage of women with low-grade cervical lesions--HPV mRNA testing versus repeat cytology. PLoS One.

[65] Persson M (2014). Triage of HR-HPV positive women with minor cytological abnormalities: a comparison of mRNA testing, HPV DNA testing, and repeat cytology using a 4-year follow-up of a population-based study. PLoS One.

[66] Liu L (2017). Role of E6/E7 mRNA in discriminating patients with high-risk human papilloma virus-positive associated with cytology-negative and atypical squamous cells of undetermined significance. Biomedical Research (India).

[67] Organization, P.A.H. HPV Tests For Cervical Cancer Screening [cited 2019 2 Feb]; Available from: https://www.paho.org/hq/index.php?option=com_content&view=article&id=11925:hpv-tests-for-cervical-cancer-screening&Itemid=41948&lang=en.

[68] Pan C (2018). Development and validation of a multiplex reverse transcript real-time PCR for E6/E7 mRNA detection of high-risk human papillomavirus. J Med Microbiol.

[69] Frega A (2016). Expression of E6/E7 HPV-DNA, HPV-mRNA and colposcopic features in management of CIN2/3 during pregnancy. Eur Rev Med Pharmacol Sci.

[70] Ca L (2012). High risk HPV DNA subtypes and E6/E7 mRNA expression in a cohort of colposcopy patients from northern Italy with high-grade histologically verified cervical lesions. Am J Transl Res.

[71] Sørbye SW (2013). HPV mRNA testing in triage of women with ASC-US cytology may reduce the time for CIN2+ diagnosis compared with repeat cytology. Curr Pharm Des.

[72] Mockel J (2011). Human papillomavirus E6/E7 mRNA testing has higher specificity than liquid-based DNA testing in the evaluation of cervical intraepithelial neoplasia. Anal Quant Cytol Histol.

[73] Frega A (2011). Prognostic implication of high risk human papillomavirus E6 and E7 mRNA in patients with intraepithelial lesions of the cervix in relationship to age. Int J Immunopathol Pharmacol.

[74] Basu P (2016). Sensitivity of APTIMA HPV E6/E7 mRNA test in comparison with hybrid capture 2 HPV DNA test for detection of high risk oncogenic human papillomavirus in 396 biopsy confirmed cervical cancers. J Med Virol.

[75] Gustinucci D (2016). Use of cytology, E6/E7 mRNA, and p16INK4a-Ki-67 to define the management of human papillomavirus (HPV)-positive women in cervical cancer screening. Am J Clin Pathol.

[76] Yang L (2017). The clinical application of HPV E6/E7 mRNA testing in triaging women with atypical squamous cells of undetermined significance or low-grade squamous intra-epithelial lesion pap smear: a meta-analysis. J Cancer Res Ther.

[77] Bruno MT (2018). A prospective study of women with ASCUS or LSIL pap smears at baseline and HPV E6/E7 mRNA positive: a 3-year follow-up. Epidemiol Infect.

