# Supplementary material for: HPV E6/E7 mRNA test for the detection of high grade cervical intraepithelial neoplasia (CIN2+): a systematic review
Source: Infect Agent Cancer. 2020 Feb 7;15:9. doi: 10.1186/s13027-020-0278-x (PMC7006188; doi:10.1186/s13027-020-0278-x)
Supplement: Supplementary file 1 — Additional file 1. Search strategy [file 13027_2020_278_MOESM1_ESM.docx]

**Supplement 1: Search strategy**

| PubMed | Scopus |
| --- | --- |
| ((((Human Papillomaviruses[Title/Abstract]) OR HPV[Title/Abstract]) AND E6/E7[Title/Abstract]) AND mRNA[Title/Abstract]) AND Cervical Intraepithelial Neoplasia[Title/Abstract] | ( TITLE-ABS-KEY ( human  AND papillomavirus )  OR  TITLE-ABS-KEY ( hpv )  AND  TITLE-ABS-KEY ( e6/e7 )  AND  TITLE-ABS-KEY ( mrna )  AND  TITLE-ABS-KEY ( cervical  AND intraepithelial  AND neoplasia ) )  AND  ( LIMIT-TO ( DOCTYPE ,  "ar" ) )  AND  ( LIMIT-TO ( LANGUAGE ,  "English" ) )  AND  ( LIMIT-TO ( SRCTYPE ,  "j" ) ) |
