# Supplementary material for: HPV E6/E7 mRNA test for the detection of high grade cervical intraepithelial neoplasia (CIN2+): a systematic review
Source: Infect Agent Cancer. 2020 Feb 7;15:9. doi: 10.1186/s13027-020-0278-x (PMC7006188; doi:10.1186/s13027-020-0278-x)
Supplement: Supplementary file 2 — Additional file 2. Risk of bias summary result: review authors’ judgements about each risk of bias item for included studies, 2011–18 [file 13027_2020_278_MOESM2_ESM.docx]

Supplement 2: Risk of bias summary result: review authors' judgements about each risk of bias item for included studies, 2011-18.

| Article | Questions to help make sense of a diagnostic test study | | | | | | | | |
| --- | --- | --- | --- | --- | --- | --- | --- | --- | --- |
|  | 1 | 2 | 3 | 4 | 5 | 6 | 7 | 8 | 9 |
| Ratnam | Y* | Y | ? | N** | Y | Y | ? | Y | Y |
| Waldstrom | Y | Y | N | ? | Y | Y | N | Y | ? |
| Fan | ? | Y | Y | ? | Y | Y | N | Y | Y |
| Han | ? | Y | Y | N | ? | Y | ? | ? | Y |
| Binnicker | Y | Y | N | ? | Y | Y | ? | Y | Y |
| Broccolo | Y | Y | ? | ? | Y | Y | N | Y | Y |
| Waldstrom | Y | Y | N | ? | Y | Y | N | Y | ? |
| Li, Y | Y | Y | N | N | Y | Y | N | Y | Y |
| Benevolo | Y | Y | N | N | Y | Y | Y | Y | Y |
| Wang | Y | Y | Y | N | Y | Y | N | Y | Y |
| Liu | Y | Y | Y | ? | Y | Y | ? | Y | Y |
| Iftner | Y | Y | Y | ? | ? | Y | N | Y | Y |
| Sorbye | ? | Y | Y | N | Y | Y | Y | Y | Y |
| Duvlis | Y | Y | Y | ? | Y | Y | N | Y | Y |
| Castro | Y | Y | N | N | ? | Y | N | Y | Y |
| Pierry | Y | Y | Y | ? | ? | Y | N | N | Y |
| Alaghehbandan | Y | Y | Y | N | Y | Y | N | Y | ? |
| Clad | Y | Y | Y | ? | Y | Y | Y | ? | Y |
| Varnai | ? | Y | Y | N | Y | Y | Y | Y | Y |
| Coquillard | Y | Y | ? | ? | Y | Y | N | Y | Y |
| Shen | Y | Y | Y | N | Y | Y | ? | Y | Y |
| Li | Y | Y | Y | ? | Y | Y | ? | Y | ? |
| Liu | Y | Y | Y | Y | Y | Y | ? | Y | ? |
| Benevolo | ? | Y | Y | ? | Y | Y | N | Y | Y |
| Liu | ? | Y | Y | N | Y | Y | N | Y | Y |
| Persson | Y | Y | N | ? | Y | Y | ? | Y | Y |
| Sorbye | ? | Y | Y | N | Y | Y | N | Y | Y |
| Andersson | Y | Y | Y | ? | Y | Y | N | N | Y |
| Oliveira | Y | Y | Y | ? | Y | Y | N | ? | Y |

Y*= Yes N**= No ?= Can’t Tell.

1Was there a clear question for the study to address? 2 Was there a comparison with an appropriate reference standard? 3Did all patients get the diagnostic test and reference standard? 4Could the results of the test have been influenced by the results of the reference standard? 5 Is the disease status of the tested population clearly described? 6Were the methods for performing the test described in sufficient detail? 7 Can the results be applied to your patients/the population of interest? 8Can the test be applied to your patient or population of interest? 9Were all outcomes important to the individual or population considered?
